# Surreptitious sympatry: Exploring the ecological and genetic separation of two sibling species

**DOI:** 10.1002/ece3.2774

**Published:** 2017-02-12

**Authors:** Line S. Cordes, Gregory O'Corry‐Crowe, Robert J. Small

**Affiliations:** ^1^ School of Ocean SciencesCollege of Natural SciencesBangor UniversityMenai BridgeUK; ^2^ Harbor Branch Oceanographic InstituteFlorida Atlantic UniversityFort PierceFLUSA; ^3^ Division of Wildlife ConservationAlaska Department of Fish and GameJuneauAKUSA

**Keywords:** climate change, dive behavior, habitat utilization, hybridization, interspecific competition, range shift

## Abstract

Climate change is having profound impacts on animal populations, and shifts in geographic range are predicted in response. Shifts that result in range overlap between previously allopatric congeneric species may have consequences for biodiversity through interspecific competition, hybridization, and genetic introgression. Harbor seals (*Phoca vitulina*) and spotted seals (*Phoca largha*) are parapatric sibling species and areas of co‐occurrence at the edges of their range, such as Bristol Bay, Alaska, offer a unique opportunity to explore ecological separation and discuss potential consequences of increased range overlap resulting from retreating sea ice. Using telemetry and genetic data from 14 harbor seals and six spotted seals, we explored the ecological and genetic separation of the two species by comparing their utilization distributions, distance from haul‐out, dive behavior (e.g., depth, duration, focus), and evidence of hybridization. Firstly, we show that harbor and spotted seals, which cannot be visually distinguished definitively in all cases, haul‐out together side by side in Bristol Bay from late summer to early winter. Secondly, we observed subtle rather than pronounced differences in ranging patterns and dive behavior during this period. Thirdly, most spotted seals in this study remained close to shore in contrast to what is known of the species in more northern areas, and lastly, we did not find any evidence of hybridization. The lack of distinct ecological separation in this area of sympatry suggests that interspecific competition could play an important role in the persistence of these species, particularly if range overlap will increase as a result of climate‐induced range shifts and loss of spotted seal pagophilic breeding habitat. Our results also highlight the added complexities in monitoring these species in areas of suspected overlap, as they cannot easily be distinguished without genetic analysis. Predicted climate‐induced environmental change will likely influence the spatial and temporal extent of overlap in these two sibling species. Ultimately, this may alter the balance between current isolating mechanisms with consequences for species integrity and fitness.

## 1 Introduction

Reproductive isolation and divergent natural selection are often central elements in speciation (Mayr, 1970; Schluter, 2009). When the geographic ranges of closely related or similar species overlap, interspecific competition may increase adaptive divergence and maintain reproductive isolation (Brown & Wilson, 1956; Grant & Grant, 2006; Pfennig & Pfennig, 2009). Alternatively, in species where reproductive barriers are not absolute, range overlap may result in successful hybridization that facilitates genetic introgression and phenotypic convergence, which can lead to a loss of unique adaptations and the emergence of new genotypes and phenotypes with different fitness (Grant et al., 2004; Lancaster, Goldsworthy, & Sunnucks, 2007). Latitudinal and elevational climate‐induced range shifts have recently been observed in a variety of species worldwide, and future climate scenarios predict further distribution shifts (Parmesan, 2006). Such shifts may create geographic overlap between previously allopatric species, resulting in increased interspecific competition and potential “hybrid zones” in closely related species with implications for biodiversity (Garroway et al., 2010; Grant et al., 2004; Shurtliff, 2013). Recent literature indicates that hybridization among mammals is more common than previously thought (Ellington & Murray, 2015; Koen et al., 2014; Lehman et al., 1991; Schwartz et al., 2004; Shurtliff, 2013). In time this may become more apparent in polar species as a result of climate‐induced northward boundary shifts causing a “polar squeeze” whereby species ranges are condensed and more likely to overlap as a result of a reduction in available habitat (Gilg et al., 2012). Hybridization is already evident in some arctic mammals, such as polar and grizzly bears, and several arctic species considered at risk of hybridization are also listed either as threatened or endangered with extinction (Kelly, Whiteley, & Tallmon, 2010). Furthermore, polar species may suffer additional detrimental effects from retreating sea ice, resulting in habitat loss, and opening of new corridors allowing disease transfer to naïve populations (Comiso et al., 2008; Kovacs & Lydersen, 2008; Kovacs et al., 2011). The mitigation and management of these impacts therefore requires an understanding of the likely ecological or evolutionary impacts of impending climate‐induced sympatry and the broader consequences for biodiversity in polar regions (Kovacs et al., 2011).

In parapatric species, distributions overlap slightly with small contact zones of co‐occurrence. The seasonal and spatial extent of contact zones, however, may influence the balance between maintaining reproductive and phenotypic separation on the one hand and genetic introgression and convergence on the other. These contact zones, therefore, offer a valuable opportunity for exploring the potential impacts of climate‐induced range shifts in closely related species and ultimate consequences for biodiversity. Where two species have long occurred in sympatry, character displacement via interspecific competition may have taken place; however, if the sympatry is relatively new, evidence of hybridization through morphological intermediates may exist. Harbor seals (*Phoca vitulina*) and spotted seals (*Phoca largha*) are parapatric in the North Pacific and overlap in distribution at the edges of their range on the Kamchatka Peninsula (Russia), Hokkaido Island (Japan), and Bristol Bay (Alaska) (Burns, 1970; Nakagawa, Kobayashi, & Suzuki, 2009; Nakagawa et al., 2010). Spotted seals are closely associated with the sea ice of the Bering, Chukchi, and Beaufort seas for much of the year, whereas harbor seals of the North Pacific Ocean and North Atlantic Ocean haul‐out on sandbanks, mud flats, and skerries, but also on glacial ice floes in some parts of their range (Burns, 1970; Da Silva & Terhune, 1988). These two species have distinct ecologies and reproductive biology, with spotted seals pupping on sea ice up to 2 months earlier (Feb–May) than harbor seals (April–July), which give birth primarily on land (Burns, 1970). However, the two species are very similar in gross morphology and in areas of seasonal range overlap both species haul‐out on terrestrial sites. They were only recognized as two separate species in the 1970s (Burns et al., 1984; Shaughnessy & Fay, 1977), and subsequent genetic and morphological investigations have established their sibling species status (Burns, Fay, & Fedoseev, 2002; Nakagawa et al., 2009; O'Corry‐Crowe & Westlake, 1997). Nevertheless, they remain extremely difficult to distinguish from one another using only external morphological features.

Distinguishing between the two species is possible, if information is available on a suite of characteristics, for example, pelage (Shaughnessy & Fay, 1977) or dentition (Burns et al., 1984). However, distinguishing between the two species using these criteria is a qualitative, not a quantitative, process and is not definitive in all cases. The limited information available during our capture operations was insufficient to definitively distinguish the two species. As such, seals we thought to be harbor seals were captured and satellite tagged in Bristol Bay, Alaska, and a few individuals subsequently exhibited long‐distance movements, one more than 1,500 km (Bristol Bay to Chukotka, the northeast coast of Russia). Such extensive movements have not been documented for harbor seals, but are typical from what is known about spotted seal behavior from more northern regions (Boveng et al., 2009; Lowry et al., 1998, 2000). Although harbor seals have been widely studied across much of their range, less is known about the spotted seal, and no ecological studies have been conducted in any area of co‐occurrence. Furthermore, because of their morphological similarity and the paucity of information on the seasonal extent of sympatry, the degree to which both species haul‐out together is unknown.

We focussed our research during the ice‐free nonbreeding season (September to December in years 2000 and 2001) after the summer molt to facilitate tag attachment and when oceanographic conditions might predict the greatest seasonal sympatry and ecological overlap. Using a combination of molecular and telemetric techniques, we employed a multifaceted approach to compare the ecological and genetic separation of harbor and spotted seals in Bristol Bay, Alaska. Our specific objectives were to (1) determine whether the two species haul‐out together; (2) explore whether there was evidence of hybridization; and (3) investigate ecological separation in ranging patterns, utilization distributions, and dive behavior of the two species.

## 2 Methods

### 2.1 Study area and capture

Seal captures took place in Egegik and Ugashik bays, located on the north side of the Alaska Peninsula, in Bristol Bay, Alaska, which is part of the Bering Sea with a maximum depth of ~70 m. In September of 2000 and 2001, after both species were known to have molted, 20 seals (10 in each year) were captured in nets near haul‐outs within the two bays, which are ~75 km apart in southeastern Bristol Bay, and then placed in hoop nets and transferred to a research vessel for processing, that is, sex, mass (kg), tissue biopsy (small ~1 cm wedge‐shaped piece of skin at the edge of the flipper), and tag deployment (Table 1a). In each year, captured seals were equipped with satellite‐linked dive recorders (SDR, T16 model developed by Wildlife Computers) that were glued onto their mid‐dorsal surface using quick‐setting epoxy. The SDR tags measured 109 × 44 × 22 mm and weighed 143 g. The seals were released near their capture sites within 2–4 hr of capture. Animals were captured and handled under National Marine Fisheries research permits 1000 and 358–1585 issued to the ADF&G.

**Table 1:** (a) Field measurements, tag performance, and (b, c) genetic determination of the 20 seals captured in Bristol Bay, Alaska, 2000–2001

ID	Sex	Mass (kg)	Capture date	Days w/locations	Locations (*N*)	Field ID	mtDNA	Microsatellites			
								No. loci screened	No. loci scored	Assignment (*Q* value)	
										*Phoca largha*	*Phoca vitulina*
a)							b)				
PV00BB02	F	30.7	12/09/2000	47	265	*P. vitulina*	*P. largha*	9	7	.967	.033
PV00BB03	M	30.2	12/09/2000	23	136	*P. vitulina*	*P. largha*	9	7	.956	.044
PV00BB04	M	46.7	12/09/2000	50	249	*P. vitulina*	*P. largha*	9	7	.950	.050
PV00BB06	F	41.4	12/09/2000	38	120	*P. vitulina*	*P. largha*	9	7	.903	.097
PV00BB10	M	45.2	13/09/2000	15	44	*P. vitulina*	*P. vitulina*	9	7	.002	.998
PV00BB11	F	33.1	13/09/2000	16	45	*P. vitulina*	*P. largha*	9	7	.954	.046
PV00BB12	F	51.9	13/09/2000	49	171	*P. vitulina*	*P. vitulina*	9	7	.002	.998
PV00BB13	F	31.7	13/09/2000	65	390	*P. vitulina*	*P. vitulina*	9	7	.004	.996
PV00BB15	F	46.2	13/09/2000	54	203	*P. vitulina*	*P. vitulina*	9	9	.023	.977
PV00BB18	M	49.0	14/09/2000	46	169	*P. vitulina*	*P. vitulina*	9	9	.001	.999
PV01BB05	F	52.7	04/09/2001	102	473	*P. vitulina*	*P. vitulina*	9	8	.001	.999
PV01BB07	F	65.0	06/09/2001	108	739	*P. vitulina*	*P. vitulina*	9	8	.001	.999
PV01BB11	F	64.1	06/09/2001	7	42	*P. vitulina*	*P. vitulina*	9	8	.001	.999
PV01BB23	F	53.3	07/09/2001	92	378	*P. vitulina*	*P. vitulina*	9	8	.002	.998
PV01BB24	F	53.5	07/09/2001	81	299	*P. vitulina*	*P. vitulina*	9	8	.002	.998
PV01BB28	F	36.4	07/09/2001	114	1136	*P. vitulina*	*P. vitulina*	9	8	.001	.999
PV01BB35	F	50.0	08/09/2001	97	495	*P. vitulina*	*P. largha*	9	9	.996	.004
PV01BB41	M	72.9	09/09/2001	87	511	*P. vitulina*	*P. vitulina*	9	8	.002	.998
PV01BB48	M	71.9	09/09/2001	112	1016	*P. vitulina*	*P. vitulina*	9	7	.001	.999
PV01BB49	F	63.1	09/09/2001	111	858	*P. vitulina*	*P. vitulina*	9	8	.002	.998
c)											
PV00BB02							*P. largha*	20	20	.997	.003
PV00BB03							*P. largha*	20	20	.995	.005
PV00BB04							*P. largha*	20	20	.998	.002
PV00BB06							*P. largha*	20	20	.995	.002
PV00BB11							*P. largha*	20	20	.998	.002
PV01BB35							*P. largha*	20	20	.998	.002

Six seals were identified as spotted seals based on a summary of mtDNA and microsatellite assignment‐based testing of harbor and spotted seals in Alaskan, Russian, and Japanese waters where field and genetic determinations do not agree. Specifically, (b) nuclear analysis based on a set of nine microsatellite loci screened in 766 harbor and 199 spotted seals and (c) nuclear analysis based on a set of 20 microsatellite loci screened in 47 harbor seals and 23 spotted seals.

### 2.2 Genetic data

Tissue biopsy samples collected from tagged seals were preserved in EtOH, and total DNA was extracted using standard protocols (O'Corry‐Crowe, Martien, & Taylor, 2003). A 588‐base pair (bp) fragment of the mitochondrial genome was amplified and 435 bp sequenced for both light and heavy strands (see Westlake & O'Corry‐Crowe, 2002). We used mtDNA sequence data (435 bp) from over 1,400 harbor seal samples from across the North Pacific, including 1,271 from Alaska, and 247 spotted seal samples from across their range (Okhotsk Sea, Bering Sea, and Chukchi Sea) to clarify mtDNA phylogeography and dispersal patterns within and phylogenetic relationships between these two species (O'Corry‐Crowe & Westlake, 1997; O'Corry‐Crowe et al., 2003; Westlake & O'Corry‐Crowe, 2002). We also generated multilocus genotypes in nine independent hypervariable microsatellite loci (Allen et al., 1995; Coltman, Don Bowen, & Wright, 1996; Goodman, 1997) that we previously screened in 766 harbor seals and 199 spotted seals (of which 38 spotted seals and 187 harbor seals were sampled in an area of known overlap). A subset of 23 spotted seals and 47 harbor seals were also screened for polymorphism in an extended set of 20 microsatellite loci to assess genetic assignment power with respect to locus number (Table S1).

### 2.3 Satellite telemetry

The SDR tags transmitted radio signals to Service Argos receivers on board National Oceanic and Atmospheric Administration polar‐orbiting satellites. The signals were processed by Service Argos to estimate locations of the tagged seals, and the precision (location quality) of the locations was provided by Service Argos based on the number of signals received (Vincent et al., 2002; see Appendix S1). The tags also transmit histogram files, which contain information on dive depth, dive duration, and time at depth (see Appendix S1). These data were recorded as number of dives or proportion of time spent in 10 bins of differing depth or duration within 6‐hr intervals. For our analyses, we derived mean and max dive depth, mean and max dive duration, dive focus and focal depth from these dive bin data (see Appendix S1).

**Figure 1:**
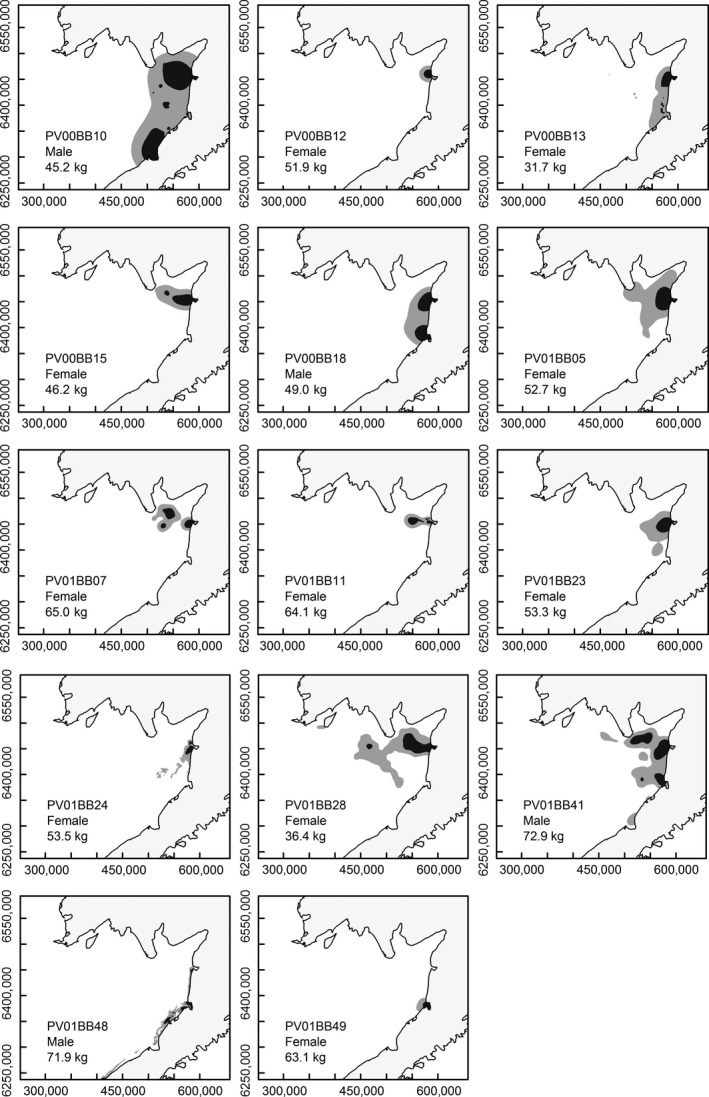
Kernel Brownian bridge 50% (dark gray) and 90% utilization distribution (light gray) for individual harbor seals

Our comparison of movements and dive behavior for the two species was focussed on the September–December period, when data were available from at least two individuals of both species. This allowed a more direct comparison of movements and dive behavior, and avoided periods when the behavior of individuals may be influenced by the approaching breeding season or periods when spotted seals are more likely to be associated with sea ice offshore. During our selected study period, one seal (PV00B02) left the Bristol Bay study area and moved into Russian waters (>1,500 km; see Figure 1). This individual was not included in subsequent analyses of movements and dive behavior as its journey into Russian waters no longer reflected a utilization distribution or distance from haul‐out, and any differences between this and other individuals in dive behavior may simply be an artifact of different habitat characteristics encountered by the individual seals.

### 2.4 Statistical analysis

#### 2.4.1 Genetic analysis

We analyzed molecular data using phylogenetic reconstruction and likelihood‐based clustering and assignment methods. Phylogenetic relationships among mtDNA sequences were inferred using maximum parsimony analysis in PAUP 4.0 (Swofford, 2002) and median joining networks, using Network 4.6 (Fluxus technology Ltd. 2011). We used the model‐based clustering algorithm, structure (v.2.3.4, Pritchard, Stephens, & Donnelly, 2000), to assess nDNA subdivision among spotted and harbor seals, assign tagged seals to species, and assess the likelihood of admixed ancestry for each animal; the method uses a Bayesian approach to estimate the most likely number of population clusters, *K*, given the data. Both admixture and no‐admixture models were applied. We used MCMC methods to integrate over the parameter space and multiple (*n* = 10) long runs with different starting conditions were conducted and summary statistics monitored for convergence. The clear species clustering meant that prior information on sample group (i.e., LOCPRIOR model; Hubisz et al., 2009) was not needed to resolve species structure.

### 2.5 Satellite telemetry and location error

There are a number of challenges associated with analyzing Argos data, which include handling location error, irregular time intervals, and dive data collected in discrete depth and duration bins. In terms of location error, >50% of locations were associated with low‐quality codes (0, A, B, Z) and simply discarding these data can severely reduce sample size and information content (Freitas et al., 2008). Furthermore, locations with high‐quality codes (1, 2, 3) only have a ~68% probability of being within the defined distances (Freitas et al., 2008). To eliminate improbable locations, yet without discarding excessive amounts of data, we applied the speed–distance–angle (SDA) filter in R (argos filter package; Freitas, 2012; R Core Team 2014), which first removes all locations with a quality code Z. Locations with swim speeds >2.5 m/s are then also removed (Dietz et al., 2013), unless the location was within 5 km of the previous one; this retains good‐quality locations in which high swim speeds are purely due to locations being recorded in quick succession. Finally, we discarded locations with unlikely turn angles, defined as all locations requiring turning angles higher than 165° and 155°, if the track prior to the location was >2.5 km and >5 km, respectively (Freitas et al., 2008). Overall, the SDA filter discarded 1,778 locations, ~18.6% of the dataset, the majority of which had poorer quality codes; B = 58.6%, A = 18.0%, 0 = 14.2%, 1 = 6.2%, 2 = 2.1%, and 3 = 0.8%. The filtered data contained 7,739 locations with the following quality codes: B = 35.3%, A = 26.2%, 0 = 8.6%, 1 = 15.2%, 2 = 9.1%, and 3 = 5.6%.

### 2.6 Utilization distribution and overall range

The kernel Brownian bridge approach, which accounts for serial autocorrelation between relocations, was used to estimate monthly UDs of individual seals (adehabitatHR package; Bullard, 1999; Calenge, 2006; Horne et al., 2007). This approach takes into account the path between two successive relocations, which may not be linear, and estimates the density probability that this path passed through any point of the study area while accounting for a certain amount of inaccuracy. Specifically, the Brownian bridge is estimated using two smoothing parameters, sig1 (related to the speed of the animal) and sig2 (related to the inaccuracy of relocations). As no declared measurement error was provided for most of the low‐quality codes, and due to the inaccuracy of the reported Argos error for locations with high‐quality codes, we assigned error measurements (sig2) based on the 68th percentile estimated error from GPS double‐tagging experiments in Costa et al. (2010) (3 = 0.5; 2 = 1; 1 = 1.2; 0 = 4.2; A = 6.2; B = 10.3 km). Sig1 for individual seals was then estimated using the liker function, which uses a maximum likelihood approach. From the kernel Brownian bridge analysis, we extracted the 50% and 90% monthly UDs for all individuals and subtracted the area of intersection with the Alaska landmass polygon. The frequency distribution of 50% and 90% monthly UDs was skewed toward smaller areas and was therefore log‐transformed.

We analyzed the 50% and 90% monthly UDs separately using generalized linear mixed models (nlme package; Pinheiro et al., 2014), which included the null model, single‐parameter models including species, month, sex, mass, a species–month interaction, as well as two, three, and four parameter additive models. The frequency distributions of monthly UDs were skewed toward smaller areas, and these data were therefore log‐transformed. Model selection was carried out using AICc scores and AICc weights. Model averaging (AICcmodavg package; Mazerolle, 2015) of the top models that accounted for 95% of the AICc weight was used to extract the β‐estimates and their 95% confidence intervals of individual parameters. We considered β‐estimates with confidence intervals that did not (or only marginally) overlap zero to have a significant effect.

### 2.7 Distance from haul‐out

To explore differences in movement, we extracted the linear distances between last haul‐out location and (1) each at‐sea location in the subsequent at‐sea period, and (2) the single at‐sea location at the maximum distance away. All distances were analyzed using generalized linear mixed modeling (Pinheiro et al., 2014) and model averaging (as described above). To account for autocorrelation in distances from haul‐out (i.e., if one location is far away from the haul‐out site, chances are that subsequent locations also are far away), the corAR1 function was applied. Furthermore, the frequency distribution of distances from haul‐out was skewed toward shorter distances, and these data were therefore log‐transformed. We ran a null model, single‐parameter models of species, sex, mass, and month, as well as two and three parameter additive models.

### 2.8 Dive behavior

The total number of dives within each bin (as described above) was multiplied by the median depth or duration value for each bin and then divided by the sum of all dives to calculate mean dive depths and durations (Folkow & Blix, 1999; see Appendix S1). Maximum depths and dive durations were based on the upper value of the bin in which the maximum depth or duration was recorded during any 6‐hr interval. Dive focus was calculated as the sum over all depth bins of the proportion of dives that fell within each bin; a finite correction factor was included that allowed this index to be used for small sample sizes (Frost, Simpkins, & Lowry, 2001):

$$\text{Dive focus}=\sum_{i=1}^{10}\left\{\frac{\left[n_{i}\left(n_{i}-1\right)\right]}{\left[N(N-1\right]}\right\}$$

where *n*_*i*_ is the number of dives in depth bin *i* and *N* is the total number of dives. Dive focus >0.50 indicates that dives within a 6‐hr period were primarily to one particular depth bin. Dive focus values were constrained between 0 and 1; thus, a logit transformation was used in the generalized linear mixed model (GLMM). The focal depth was defined as the dominant dive bin within which dive focus was >0.50. The different dive behaviors were analyzed using GLMM and model averaging, as described above. We used the corCAR(~time|ID) function to account for autocorrelation and unequal time spacing between 6‐hr intervals among repeated measures of individual seals. We ran a null model as well as single and additive models including a combination the parameters species, month, sex, mass, and a species–month interaction.

## 3 Results

### 3.1 Genetic analyses

Earlier phylogenetic studies determined that harbor and spotted seals were reciprocally monophyletic for mtDNA (O'Corry‐Crowe & Westlake, 1997). Of the 20 seals tagged in our study, 14 had mtDNA haplotypes characteristic of harbor seals, whereas six had maternal lineages characteristic of spotted seals (Table 1b). To assess whether the mtDNA “miss‐assignments” represented (1) misidentifications in the field; (2) incomplete mtDNA lineage sorting, or (3) hybridization, we compared our findings to reference datasets of 766 North Pacific harbor seals and 199 spotted seals from across the species’ ranges that had complete or near complete genetic profiles for both mtDNA and the nine microsatellite loci screened in both species (Table 1b). Defining known harbor and spotted seals as animals sampled in areas of allopatry, we confirmed that mtDNA is reciprocally monophyletic across these two species. Bayesian cluster analysis, even allowing for admixture (MCMC bur‐in of 50,000, followed by 1 × 10^6^ reps, no LOCPRIOR), also clearly differentiated two discrete genetic clusters (*K *=* *2, Pr(2/*X*) ≈ 1.0) based on the nDNA data that are consistent with harbor and spotted seals. Furthermore, no evidence of mixed ancestry that may indicate recent hybridization has been documented at the nuclear loci to date. All reference seals had high (*Q* > 0.81) assignment probabilities to one species.

In all cases, the nuclear DNA agreed with the mtDNA data in species assignment: All six tagged seals found to possess a spotted seal mtDNA lineage were unambiguously assigned to the spotted seal genetic cluster (allowing for admixture, *Q* > 0.9, Table 1b). Furthermore, all seals with harbor seal mtDNA were assigned to *P. vitulina* for nDNA (Table 1b). Whereas the nine‐locus microsatellite dataset yielded very strong assignments to one species or the other (Table 1b), we were concerned that the number of independent loci screened may not be sufficient for unambiguous assignments or estimation of mixed ancestries. Therefore, we ran a subset of 70 seals, including the six tagged seals assigned to *P. largha*, for a total of 20 independent microsatellite loci (Table 1c). Apart from slightly higher ancestry likelihoods for the most likely species, the results were similar to the analysis that used the lower number of loci (Table 1c).

### 3.2 Utilization distributions and distance from haul‐out

**Figure 2:**
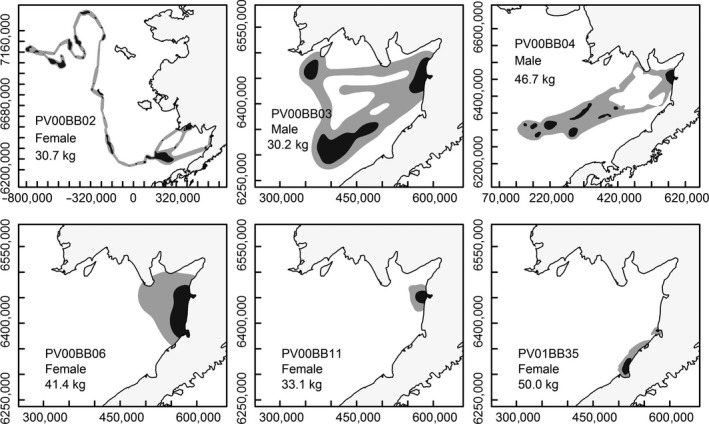
Kernel Brownian bridge 50% (dark gray) and 90% utilization distribution (light gray) for individual spotted seals

Overall, there was substantial individual variation in utilization distributions and movement patterns (Figures 1 and 2). However, the largest UDs (both 50% and 90% utilization distributions) and longest distances travelled between haul‐outs to at‐sea locations were recorded for spotted seals (Figures 1 and 2); these also exhibited the greatest variation.

**Figure 3:**
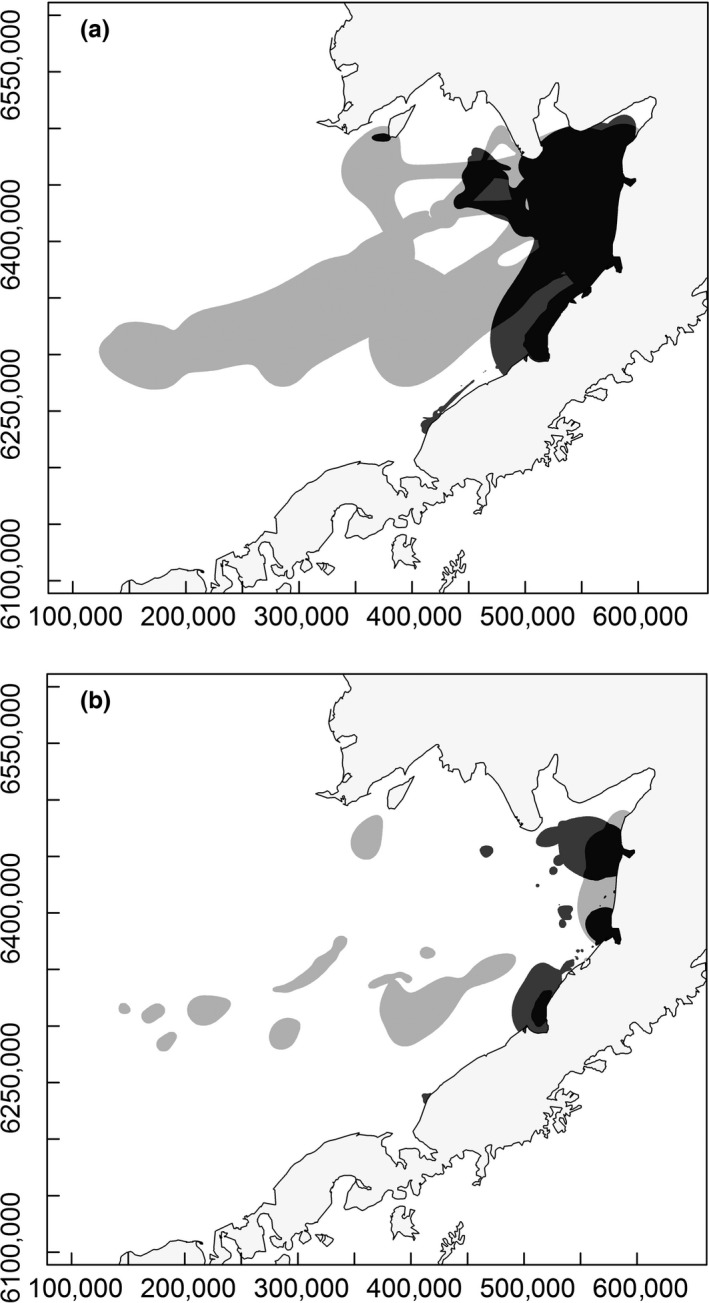
Kernel Brownian bridge (a) 90% and (b) 50% utilization distributions for all harbor seals (medium gray) and spotted seals (light gray) with the areas of overlap indicated in dark gray

For the GLMMs of the 50% and 90% monthly UDs, the top model in both cases accounted for most of the weight and did not include species as a parameter (see Table S2). Model averaging did not reveal any species difference in the size of the 50% or 90% monthly UDs, but there were significant differences in the size of UDs across months, with UDs in September being the smallest, and UDs in December being the largest. There was also a negative relationship between monthly UD and mass, and monthly UDs were larger for males compared to females (Table 2). The area of overlap between the species’ 50% UD covered 35% of the harbor seal 50% UD and 36% of the spotted seal 50% UD (Figure 3), while the area of overlap between the species’ 90% UD covered 69% of the harbor seal 90% UD and 36% of the spotted seal 90% UD (Figure 3).

**Table 2:** β‐estimates of model parameters for the utilization distribution analyses with 95% confidence intervals

Model parameters	50% UDs		90% UDs	
	β	95% CI	β	95% CI
Species (*Phoca vitulina*)	−0.77	−1.82/0.29	−0.75	−1.78/0.28
Month (Oct)	**0.62**	0.32/0.92	**0.46**	0.18/0.73
Month (Nov)	**0.51**	0.21/0.82	**0.40**	0.12/0.68
Month (Dec)	**1.02**	0.71/1.34	**0.80**	0.51/1.09
Sex (male)	**0.91**	0.10/1.71	**1.30**	0.50/2.10
Mass	−**0.04**	−0.07/−0.01	−**0.05**	−0.08/−0.01

Confidence intervals that do not overlap zero signify a significant effect/difference and are highlighted in bold.

Neither of the analyses of linear distances from haul‐out to subsequent at‐sea locations and maximum distance to haul‐out during an at‐sea bout (GLMM) showed evidence of a species‐specific difference (Tables S2 and 3). Instead, linear distance to haul‐out generally increased across months, and was larger for males compared to females. For maximum distance to haul‐out, distances in October and December were significantly larger than in September. Both linear distance and maximum distance were larger for lighter individuals compared to heavier ones (Table 3).

**Table 3:** β‐estimates of model parameters for the movement and dive behavior analyses with 95% confidence intervals

Model parameters	Dist. from haul‐out		Max dist. from haul‐out		Dive focus		Focal depth	
	β	95% CI	β	95% CI	β	95% CI	β	95% CI
Species (*Phoca vitulina*)	−0.38	−1.06/0.31	−0.03	−0.49/0.44	**0.04**	0.01/0.07	0.33	−8.46/9.12
Month (Oct)	**0.39**	0.21/0.57	**0.43**	0.05/0.81	**0.01**	0.00/0.02	**5.17**	2.44/7.91
Month (Nov)	**0.60**	0.39/0.81	0.37	−0.04/0.77	0.00	−0.01/0.01	**4.54**	1.73/7.34
Month (Dec)	**0.85**	0.63/1.07	**0.49**	0.03/0.96	**0.01**	0.00/0.02	**12.28**	9.48/15.07
Sex (male)	**0.68**	0.12/1.24	0.24	−0.14/0.62	−**0.04**	−0.07/−0.02	**7.89**	0.60/15.19
Mass	−**0.02**	−0.04/0.00	−**0.02**	−0.03/0.00	0.00	0.00/0.00	−0.18	−0.45/0.10
Species (*vit*):Month (Oct)	−0.08	−0.49/0.34	−0.72	−1.68/0.23	0.01	−0.01/0.04	−2.18	−9.42/5.06
Species (*vit*):Month (Nov)	−0.15	−0.69/0.40	0.21	−0.08/1.23	−0.01	−0.04/0.03	−**9.51**	−17.46/−1.56
Species (*vit*):Month (Dec)	0.09	−0.53/0.70	0.22	−0.86/1.30	**0.03**	0.00/0.07	−2.56	−10.86/5.73

Model parameters	Mean dive depth		Max dive depth		Mean dive duration		Max dive duration	
	β	95% CI	β	95% CI	β	95% CI	β	95% CI
Species (*Phoca vitulina*)	1.39	−3.66/6.44	−1.71	−9.64/6.22	0.29	−0.14/0.72	1.22	−0.12/2.56
Month (Oct)	**3.49**	2.14/4.85	**5.20**	3.12/7.27	**0.16**	0.06/0.26	**0.17**	−0.13/0.48
Month (Nov)	**3.39**	1.99/4.80	**5.62**	3.46/7.78	**0.28**	0.17/0.38	**0.16**	−0.15/0.48
Month (Dec)	**8.86**	7.46/10.25	**12.65**	10.49/14.80	**0.73**	0.62/0.83	**0.27**	−0.04/0.58
Sex (male)	2.95	−1.66/7.56	**7.58**	0.57/14.58	−0.09	−0.50/0.31	−0.44	−1.76/0.89
Mass	2.92	−1.69/7.53	−0.01	−0.37/0.17	**0.01**	0.00/0.03	0.03	−0.02/0.09
Species (*vit*):Month (Oct)	−0.82	−4.10/2.47	−2.55	−7.52/2.43	−0.11	−0.36/0.14	0.73	−0.02/1.48
Species (*vit*):Month (Nov)	−**4.59**	−8.33/−0.86	−**8.83**	−14.48/−3.17	−0.15	−0.43/0.12	0.30	−0.53/1.13
Species (*vit*):Month (Dec)	−0.05	−3.80/3.70	−5.24	−10.93/0.46	0.20	−0.07/0.48	0.30	−0.53/1.13

Confidence intervals that do not overlap zero signify a significant effect/difference and are highlighted in bold.

### 3.3 Dive behavior

**Figure 4:**
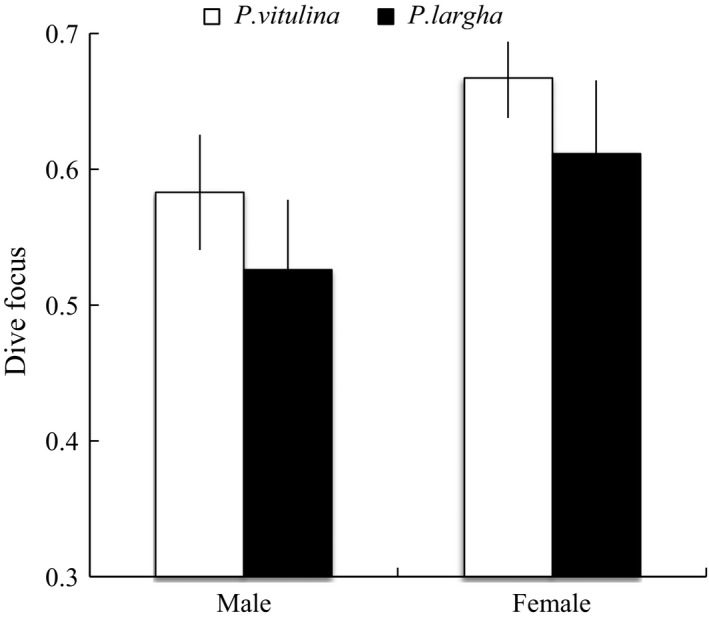
Species‐ and sex‐specific predicted estimates of dive focus (95% CI) from the top model for an individual of average mass

As with movement patterns, we recorded a wide range of dive behaviors in both harbor and spotted seals. The analysis of dive focus (GLMM) revealed six top models with ΔAICc < 2, which accounted for 0.95 of the AICc weight (see Table S3). Although both species were very focussed in their dives (>0.50), harbor seals were significantly more focussed compared to spotted seals (Table 3, Figure 4). Furthermore, males appeared less focussed in their dives compared to females (Table 3, Figure 4). For focal depth, the GLMM revealed seven top models with ΔAICc < 3, accounting for 0.96 of the AICc weight (see Table S3). Model averaging indicated that focal depth increased across months and that there was a species‐specific differences in focal depth in November. Males also had a deeper focal depth compared to females (Table 3).

For mean dive depth, there were seven models within ΔAICc < 3, which accounted for 0.97 of the AICc weight (Table S3). Again, model averaging did not reveal any species or sex‐specific differences in mean dive depth; however, as above, mean dive depth increased across months and there was a species‐specific difference in focal depth in November (Table 3; see similar results for maximum dive depth in Tables S3 and 3). For mean dive duration, there were six models with ΔAICc < 5, accounting for 0.96 of the AICc weight (Table S3). Again, model averaging did not reveal a significant species difference in mean dive duration, despite species being a parameter in the top model; however, there was a general increase in mean dive duration across months (Table 3; see results for maximum dive duration in Tables S3 and 3).

## 4 Discussion

All of the 20 seals captured in Bristol Bay were initially identified as harbor seals during field operations, but the long‐distance movement by PV00BB02 was not typical of the species, and thus prompted further investigation. Genetic analysis revealed that six of the 20 seals caught were actually spotted seals. These findings not only emphasize the strong morphological similarity of these two phocids, but also reveal their tendency to haul‐out together at a number of discrete coastal sites in late summer, autumn, and early winter. Satellite tracking documented individual variation in ranging patterns and dive behavior, gender and size‐specific differences in habitat utilization, and a temporal trend toward longer range movements and longer, deeper dives from late summer to early winter. Interestingly, this study did not reveal dramatic species differences in movement and dive behavior in this area of seasonal sympatry, apart from a subtle difference in dive focus. We did, however, find that some spotted seals tended toward more expansive movements further from shore. These generally consisted of just a few trips, hence the nonuniform kernels, and were most likely outweighed by the higher frequency of shorter movements. Nevertheless, this may reflect subtle differences in foraging strategy between the two species at a time of year when they co‐occur in the southeast Bering Sea, and highlight what may be greater flexibility and range in spotted seal movements compared to that of harbor seals. More substantial species differences may occur in the late winter or early spring when the sea ice reaches Bristol Bay or when spotted seals may travel further offshore to locate breeding habitat around the sea ice. During both winters, sea ice did not reach Bristol Bay until December/January (National Snow & Ice Data Center); thus, the behavior of individual seals was unlikely to be significantly affected by this seasonal phenomenon during our study period (apart from PV00BB02 which may have been associated with sea ice in Russian waters).

The at‐sea movements and dive behavior of harbor seals have been widely studied across the temperate regions of the northern hemisphere (e.g., Bjørge et al., 1995; Boness, Bowen, & Oftedal, 1994; Eguchi & Harvey, 2005; Frost et al., 2001; Thompson et al., 1998; Tollit et al., 1998) and concur with results of our study, whereby individuals typically remained within 70 km of haul‐out sites, dive durations were 2–4 min, and dive depths were <25 m (dependent on bathymetry). However, substantially greater dive depths and durations of up to 480 m and 35 min have also been recorded (Eguchi & Harvey, 2005). Current information on spotted seal movements has been obtained primarily from more northerly areas (Chukchi and Bering seas), which are highly influenced by seasonal sea ice. Seals first haul‐out on sea ice in October or November and remain associated with the sea ice through June (Boveng et al., 2009; Lowry et al., 1998, 2000). Spotted seals tagged in the Chukchi Sea during a time period similar to our study often undertook long‐distance trips to sea (~1,000 km) lasting more than 30 days (Lowry et al., 2000), similar to the behavior of PV00BB02 in this study. In the northern Bering Sea between August and October, spotted seals remained closer to shore south of the sea ice edge, while later in the winter (January onwards), seals were typically located further offshore either on or north of the edge of the sea ice (Lowry et al., 1998).

Despite the fact that the two species could not be visually distinguished in the field and exhibited many similarities in movement and dive behavior, the genetic analysis did not reveal any evidence of hybridization. The small sample size of animals in this study, however, cannot exclude the possibility of interbreeding between these sibling species. However, the absence of documented mixed ancestry in the much larger reference sample sets that were genotyped for both species is noteworthy. Differences in breeding season and in preferred breeding habitat likely limit opportunities for interbreeding. Age‐specific segregation has been observed in spotted seals in Japan whereby immature seals are typically found at the southern edge of their distribution (Mizuno, Suzuki, & Ohtaishi, 2001). The possibility of hybridization may thus be reduced further if the Alaskan spotted seal population was segregated by age. In fact, most spotted seals in our study were likely juveniles, based on their mass at capture (range 30.2–50.0 kg; mean 38.7 kg; Boveng et al., 2009).

Although we developed our study retrospectively after documenting the long‐distance movement of PV00BB02, our data represent a rare opportunity to investigate the ecological separation of two parapatric sibling species, and to consider the potential consequences of changes in range overlap that may result from climate change. In comparison, studies of other sympatric pinnipeds, such as Steller sea lions and northern fur seals, have shown very distinct ecological separation occupying clearly different niches, that is, near‐shore benthic forager versus offshore pelagic forager (Waite et al., 2012). Furthermore, ecological separation within a single species (e.g., northern fur seal; Robson et al., 2004) has also been shown to be more dramatic than the differences between harbor and spotted seals in our study. Harbor and spotted seals in Bristol Bay currently appear to haul‐out together and, overall, have very similar ecologies during this period. Nevertheless, introgression is likely rare as there may be little overlap during the breeding season when spotted seals most likely are located further offshore along the edge of the sea ice. From the data that were available during late winter (i.e., after December), one of three spotted seals did conduct longer distance movements, although it returned to Bristol Bay. In other regions, spotted seals have been observed to spend part of the year feeding in one area before returning to breeding grounds elsewhere (Won & Yoo, 2004), which again would reduce the possibility of interbreeding if this was the case for the spotted seals in Bristol Bay.

Isolating mechanisms that maintain species integrity among sibling species typically involve allopatry, phenotypic divergence, or both (Mayr, 1970). In allopatric species, the extent of phenotypic divergence may be limited if both species occupy similar but geographically isolated niches. In contrast, closely related species whose ranges overlap substantially often occupy distinct niches and display greater phenotypic divergence (Grant & Grant, 2006; Lack, 1983; Schluter, Price, & Grant, 1985). Where the range of two similar species overlap, the degree of phenotypic divergence or character displacement, including morphology, breeding behavior, or ecological differences, is driven by the level of interspecific competition, which is expected to be more intense in areas of range overlap (Brown & Wilson, 1956; Grant & Grant, 2006). Thus, environmental changes that alter the degree of range and niche overlap among closely related species might be expected to also alter the ecological and reproductive relationship which may result in increased divergence on the one hand or a breakdown of species barriers on the other. Such environmental change may also increase species extinction risk as the competitive exclusion of one species by another becomes more widespread due to an increase in the extent of range and niche overlap. Under such a scenario, a more temperate subarctic species may be favored. Extinction probabilities of one species in such a manner, however, could be offset by its ability to adapt.

In this study, we documented two morphologically similar species that are currently maintaining genetic distinctness despite an apparent limited niche separation during the season of sympatry (late summer to early winter). This contrasts with extensive niche separation at other locations and at other times of the year, including the separation of breeding season and habitat (sea ice vs. coastal areas). In the Arctic and subarctic, changes to the cryosphere are already altering species distributions, behavior, and ecology (see Gilg et al., 2012). Such climate‐induced changes in the environment will likely influence the spatial and temporal extent of range and ecological overlap of spotted and harbor seals. We contend this may alter the delicate balance between current isolating mechanisms, including competitive exclusion, asynchronous breeding, and genetic introgression with consequences for species integrity and fitness. Predicting the effects of climate change on biodiversity is one of the most pressing eco‐evolutionary challenges (Thomas et al., 2004) and requires a detailed understanding of species’ ecology and habitat use, an understanding hindered when co‐occurring sibling species are not visually distinguishable.

## Conflict of Interest

None declared.
