# Supplementary material for: Surreptitious sympatry: Exploring the ecological and genetic separation of two sibling species
Source: Ecol Evol. 2017 Feb 12;7(6):1725–36. doi: 10.1002/ece3.2774 (PMC5355204; doi:10.1002/ece3.2774)
Supplement: Supplementary file 1 [file ECE3-7-1725-s001.docx]

**Table S1.** Microsatellite loci used in this study. All loci were originally typed on pinniped species, have been optimized for use on *P. largha* and *P. vitulina* and were found to have no inherent problems (e.g. allelic dropout, null alleles) that would preclude their use in studies of population structure and inheritance in both species.

| **Microsatellite locus** | **Species** | **Reference** |
| --- | --- | --- |
| Hg4.2* | *Halichoerus grypus* | Allen *et al*., 1995 |
| Hg6.1* | *Halichoerus grypus* | Allen *et al.*, 1995 |
| Hg6.3* | *Halichoerus grypus* | Allen *et al.*, 1995 |
| Hg8.9* | *Halichoerus grypus* | Allen *et al.*, 1995 |
| Hg8.10* | *Halichoerus grypus* | Allen *et al.*, 1995† |
| Pvc19* | *Phoca vitulina* | Coltman *et al*., 1996 |
| Pvc78* | *Phoca vitulina* | Coltman *et al.*, 1996 |
| Pvc26 | *Phoca vitulina* | Coltman *et al.*, 1996 |
| Pv9* | *Phoca vitulina* | Goodman, 1997 |
| Pv11* | *Phoca vitulina* | Goodman, 1997 |
| Pv16 | *Phoca vitulina* | Goodman, 1997 |
| Aa4 | *Arctocephalus australis* | Gemmell *et al.*, 1997 |
| Hl-4 | *Hydrurga leptonyx* | Davis *et al.*, 2002 |
| Lc-28 | *Lobodon carcinophagus* | Davis *et al*., 2002 |
| Lw-7 | *Leptonychotes weddellii* | Davis *et al.*, 2002 |
| M11a | *Mirounga* sp. | Hoelzel *et al*., 2001 |
| ZcCgDh1.8 | *Zalophus californianus* | Hernandez-Velazquez *et al*., 2005 |
| ZcCgDh4.7 | *Zalophus californianus* | Hernandez-Velazquez *et al.*, 2005 |
| ZcCgDh5.8 | *Zalophus californianus* | Hernandez-Velazquez *et al.*, 2005 |
| ZcCgDh7 | *Zalophus californianus* | Hernandez-Velazquez *et al*., 2005 |
| ZcCgDhB14 | *Zalophus californianus* | Hernandez-Velazquez *et al*., 2005 |

*the nine loci used in the initial analysis of the tagged Bristol Bay seals

† locus Hg8.10 was not used in the species comparisons involving 20 loci.

Allen, P. J., Amos, W., Pomeroy, P.P. & Twiss, S.D. (1995) Microsatellite variation in grey seals (Halichoerus grypus) shows evidence of genetic differentiation between two British breeding colonies. *Molecular Ecology*, **4**, 653–662.

Coltman, D.W., Bowen, W.D. & Wright, J.M. (1996) PCR primers for harbour seal (*Phoca vitulina concolour*) microsatellites amplify polymorphic loci in other pinniped species. *Molecular Ecology*, **5**, 161-163.

Davis, C.S., Gelatt, T.S., Siniff, D. & Strobeck, C. (2002) Dinucleotide microsatellite markers from the Antarctic seals and their use in other pinnipeds. *Molecular Ecology Notes*, **2**, 203–208.

Goodman, S.J. (1997) Dinucleotide repeat polymorphism at seven anonymous microsatellite loci cloned from the European harbour seal (*Phoca vitulina vitulina*). *Animal Genetics*, **28**, 310-311.

Gemmell, N.J., Allen, P.J., Goodman, S.J. & Reed, J.Z. (1997) Interspecific microsatellite markers for the study of pinniped populations. *Molecular Ecology*, **6**, 661-666.

Hernandez-Velazquez, F.D., Galindo-Sanchez, C.E., Taylor, M.I., De La Rosa-Velez, J., Cote, M., Schramm, Y., Aurioles-Gamboa, D. & Roci, C. (2005) New polymorphic microsatellite markers for California sea lions (*Zalophus californianus*). *Molecular Ecology Notes*, **5**, 140-142.

Hoelzel, A.R., Campagna, C. & Arnbom, T. (2001) Genetic and morphometric differentiation between island and mainland southern elephant seal populations. *Proceedings of the Royal Society of London. Series B: Biological Sciences*, **268**, 325-332.
