# Supplementary material for: Surreptitious sympatry: Exploring the ecological and genetic separation of two sibling species
Source: Ecol Evol. 2017 Feb 12;7(6):1725–36. doi: 10.1002/ece3.2774 (PMC5355204; doi:10.1002/ece3.2774)
Supplement: Supplementary file 2 [file ECE3-7-1725-s002.docx]

**Table S2.** Model selection for movement analyses of seals from Bristol Bay, Alaska, 2000-2001. Only models where cumulative AICc weight ≥0.95 are presented.

|  | **K** | **ΔAICc** | **AICc weight** | **Likelihood** |
| --- | --- | --- | --- | --- |
| ***50% utilisation distribution*** |  |  |  |  |
| month + sex + mass | 8 | 0.00 | 0.43 | -53.13 |
| species + month + sex + mass | 9 | 1.71 | 0.18 | -52.60 |
| month + mass | 7 | 2.83 | 0.10 | -55.87 |
| species + month | 7 | 2.95 | 0.10 | -55.93 |
| species + month + mass | 8 | 3.16 | 0.09 | -54.71 |
| species + month + sex | 8 | 3.95 | 0.06 | -55.10 |
|  |  |  |  |  |
| ***90% utilisation distributions*** |  |  |  |  |
| month + sex + mass | 8 | 0.00 | 0.54 | -49.38 |
| species + month + sex + mass | 9 | 1.16 | 0.30 | -48.58 |
| species + month + sex | 8 | 3.86 | 0.08 | -51.31 |
| species + month | 7 | 5.56 | 0.03 | -53.49 |
|  |  |  |  |  |
| ***Distance from haul-out*** |  |  |  |  |
| month + sex + mass | 9 | 0.00 | 0.41 | -4345.09 |
| species + month + sex | 9 | 1.42 | 0.20 | -4345.80 |
| species + month + sex + mass | 10 | 1.59 | 0.19 | -4344.88 |
| species + month | 8 | 2.98 | 0.09 | -4347.58 |
| month | 7 | 3.50 | 0.07 | -4348.84 |
|  |  |  |  |  |
| ***Max distance from haul-out*** |  |  |  |  |
| month + sex + mass | 12 | 0.00 | 0.23 | -741.57 |
| sex + mass | 8 | 0.49 | 0.18 | -744.94 |
| mass | 10 | 0.64 | 0.16 | -746.04 |
| species + sex + month + mass | 3 | 1.94 | 0.09 | -741.49 |
| month | 11 | 2.39 | 0.07 | -744.85 |
| species + mass | 6 | 2.70 | 0.06 | -746.04 |
| null | 15 | 2.73 | 0.06 | -748.11 |
| species*month + mass | 13 | 3.69 | 0.04 | -740.24 |
| species + month | 5 | 3.86 | 0.03 | -744.54 |
| species | 1 | 4.33 | 0.03 | -747.89 |
| sex | 9 | 4.43 | 0.02 | -747.93 |
