# Supplementary material for: Surreptitious sympatry: Exploring the ecological and genetic separation of two sibling species
Source: Ecol Evol. 2017 Feb 12;7(6):1725–36. doi: 10.1002/ece3.2774 (PMC5355204; doi:10.1002/ece3.2774)
Supplement: Supplementary file 3 [file ECE3-7-1725-s003.docx]

**Table S3.** Model selection for dive behaviour analyses. Only models where cumulative AICc weight ≥0.95 are presented.

|  | **K** | **ΔAICc** | **AICc weight** | **Likelihood** |
| --- | --- | --- | --- | --- |
| ***Dive focus*** |  |  |  |  |
| species*month + sex | 12 | 0.00 | 0.22 | 2474.08 |
| species + sex | 6 | 0.22 | 0.20 | 2467.93 |
| species + sex + month + mass | 10 | 0.37 | 0.19 | 2471.88 |
| species + sex + month | 9 | 0.79 | 0.15 | 2470.66 |
| sex + mass | 6 | 1.53 | 0.10 | 2467.28 |
| sex + month + mass | 9 | 1.95 | 0.08 | 2470.08 |
|  |  |  |  |  |
| ***Focal depth*** |  |  |  |  |
| sex + month + mass | 9 | 0.00 | 0.24 | -5890.96 |
| species*month + sex | 12 | 0.65 | 0.18 | -5888.24 |
| month | 7 | 0.81 | 0.16 | -5893.38 |
| species + sex + month | 9 | 1.38 | 0.12 | -5891.65 |
| species + sex + month + mass | 10 | 1.75 | 0.10 | -5890.82 |
| species*month | 11 | 2.05 | 0.09 | -5889.95 |
| species + month | 8 | 2.74 | 0.06 | -5893.34 |
|  |  |  |  |  |
| ***Max dive depth*** |  |  |  |  |
| species*month + sex | 12 | 0.00 | 0.46 | -11595.75 |
| species*month | 11 | 1.69 | 0.20 | -11597.61 |
| sex + month + mass | 9 | 3.55 | 0.08 | -11600.55 |
| species*month + mass | 12 | 3.67 | 0.07 | -11597.59 |
| species + sex + month | 9 | 3.89 | 0.07 | -11600.72 |
| month | 7 | 3.94 | 0.06 | -11602.76 |
| species + sex + month + mass | 10 | 5.54 | 0.03 | -11600.54 |
|  |  |  |  |  |
| ***Mean dive depth*** |  |  |  |  |
| species*month | 11 | 0.00 | 0.25 | -10735.87 |
| month | 7 | 0.31 | 0.22 | -10740.05 |
| species*month + sex | 12 | 0.50 | 0.20 | -10735.11 |
| species*month + mass | 12 | 1.99 | 0.09 | -10735.86 |
| species + month | 8 | 2.13 | 0.09 | 10739.96 |
| species + sex + month | 9 | 2.64 | 0.07 | 10739.20 |
| sex + month + mass | 9 | 2.98 | 0.06 | 10739.38 |
|  |  |  |  |  |
| ***Max dive duration*** |  |  |  |  |
| species | 5 | 0.00 | 0.24 | -7602.64 |
| mass | 5 | 1.05 | 0.14 | -7603.17 |
| species + mass | 6 | 1.41 | 0.12 | -7602.34 |
| null | 4 | 1.77 | 0.10 | -7604.53 |
| species + sex | 6 | 1.78 | 0.10 | -7602.53 |
| sex + mass | 6 | 2.22 | 0.08 | -7602.75 |
| species + month | 8 | 3.01 | 0.05 | -7601.13 |
| sex | 5 | 3.47 | 0.04 | -7604.38 |
| month | 7 | 4.76 | 0.02 | -7603.01 |
| species + sex + month | 9 | 4.78 | 0.02 | -7601.01 |
| species*month | 11 | 5.10 | 0.02 | -7599.16 |
|  |  |  |  |  |
| ***Mean dive duration*** |  |  |  |  |
| species*month | 11 | 0.00 | 0.40 | -3717.73 |
| species*month + mass | 12 | 0.86 | 0.26 | -3717.16 |
| species*month + sex | 12 | 1.98 | 0.15 | -3717.72 |
| species + month | 8 | 3.78 | 0.06 | -3722.65 |
| month | 7 | 4.02 | 0.05 | -3723.77 |
| sex + month + mass | 9 | 4.86 | 0.04 | -3722.18 |
