# Supplementary material for: Surreptitious sympatry: Exploring the ecological and genetic separation of two sibling species
Source: Ecol Evol. 2017 Feb 12;7(6):1725–36. doi: 10.1002/ece3.2774 (PMC5355204; doi:10.1002/ece3.2774)
Supplement: Supplementary file 4 [file ECE3-7-1725-s004.docx]

**Appendix S1. Additional information on satellite telemetry**

Each Argos locations are assigned one of seven quality codes: 3=true position within 150m; 2=true position between 150 and 350m; 1= true position between 350 and 1000m; 0=true position more than 1000m from reported location; A=no estimate of accuracy as only 3 signals where received; B=no estimate of accuracy as only 2 messages where received; and Z=rejected. A sensor on the tag also records when the tag is wet or dry (i.e. whether the seal is at-sea or hauled-out). In order to avoid spurious dry recordings the tag is set to only record dry status once the tag had been dry for 10 transmissions (i.e. 7 minutes). These data on changes in wet/dry status were joined to the location data whereby any location with a timestamp falling between a dry and wet recording where logged as dry, and any location with a timestamp falling between a wet and dry recording were logged as wet. The tags also transmit histogram files, which contain information on dive depth, dive duration, and time at depth. These data are recorded as number of dives or proportion of time spent in ten bins of differing depth or duration within 6-hour intervals. Dive depth was recorded as the number of dives within the upper bin thresholds of 4, 10, 20, 36, 50, 76, 100, 150, 200, and >200m, whereas dive durations were recorded as the number of dives within the upper bin thresholds of 1, 2, 3, 4, 5, 6, 8, 10, 12, and >12mins. Finally, time at depth is recorded as proportion of time spent within the upper bin thresholds of 0, 2, 4, 10, 20, 50, 76, 100, 150, and >150m. Mean dive depth and duration were calculated from these data. For example, 20 dives in the 4 m depth bin, 10 dives in the 10 m depth bin, 30 dives in the 20 m depth bin, and no dives in the upper bins (i.e.: (20 x 2 m + 10 x 7 m + 30 x 15 m) / 60) would equate to a mean dive depth of 9.3 m. No dives were recorded in the 100, 150, 200 and >200 m depth bins, which concurs with the maximum depth of Bristol Bay (~70 m).
